# Changes in neural resting state activity in primary and higher-order motor areas induced by a short sensorimotor intervention based on the Feldenkrais method

**DOI:** 10.3389/fnhum.2015.00232

**Published:** 2015-04-28

**Authors:** Julius Verrel, Eilat Almagor, Frank Schumann, Ulman Lindenberger, Simone Kühn

**Affiliations:** ^1^Center for Lifespan Psychology, Max Planck Institute for Human DevelopmentBerlin, Germany; ^2^The Jerusalem Academy of Music and DanceJerusalem, Israel; ^3^Laboratoire Psychologie de la Perception, Université Paris DescartesParis, France

**Keywords:** sensorimotor learning, functional magnetic resonance, resting state activity, Feldenkrais method, touch, foot

## Abstract

We use functional magnetic resonance imaging to investigate short-term neural effects of a brief sensorimotor intervention adapted from the Feldenkrais method, a movement-based learning method. Twenty-one participants (10 men, 19–30 years) took part in the study. Participants were in a supine position in the scanner with extended legs while an experienced Feldenkrais practitioner used a planar board to touch and apply minimal force to different parts of the sole and toes of their left foot under two experimental conditions. In the *local* condition, the practitioner explored movement within foot and ankle. In the *global* condition, the practitioner focused on the connection and support from the foot to the rest of the body. Before *(baseline)* and after each intervention *(post-local, post-global)*, we measured brain activity during intermittent pushing/releasing with the left leg and during resting state. Independent localizer tasks were used to identify regions of interest (ROI). Brain activity during left-foot pushing did not significantly differ between conditions in sensorimotor areas. Resting state activity (regional homogeneity, ReHo) increased from *baseline* to *post-local* in medial right motor cortex, and from *baseline* to *post-global* in the left supplementary/cingulate motor area. Contrasting *post-global* to *post-local* showed higher ReHo in right lateral motor cortex. ROI analyses showed significant increases in ReHo in pushing-related areas from *baseline* to both *post-local* and *post-global*, and this increase tended to be more pronounced *post-local*. The results of this exploratory study show that a short, non-intrusive sensorimotor intervention can have short-term effects on spontaneous cortical activity in functionally related brain regions. Increased resting state activity in higher-order motor areas supports the hypothesis that the *global* intervention engages action-related neural processes.

## Introduction

The Feldenkrais method is a movement-based learning method aimed at improving organization of the body in action (Feldenkrais, 1947; Buchanan, 2012). One basic assumption of this approach is that movement variation, guided somatosensory attention, and hands-on manipulation can provide meaningful information to the nervous system by clarifying functional relationships along the body and with the environment (e.g., connection between body parts, support from the floor, movement distribution, orientation in space). In this explorative study, we investigate the role of the practitioner's focus on functional relationships between body parts in one particular Feldenkrais technique applied to the foot, the “artificial floor” (e.g., Feldenkrais, 1981), by assessing the neural effects of two subtly different forms of the manipulation. In the *local* condition, the manipulation is focused on anatomical relationships and mobility within the foot. In the *global* condition, the manipulation explores the connections from the foot to the rest of the body, focusing on the function of the foot for body support. We use functional magnetic resonance imaging (fMRI) to investigate potential short-term effects of these two forms of the artificial floor on neural activity during a functionally related motor task (gently pushing with one foot onto a horizontal support surface) as well as during resting state. We hypothesized that the *local* manipulation would lead to increased activity in primary sensorimotor areas representing the stimulated foot, while the *global* manipulation would engage more widespread and higher-level motor areas.

### The feldenkrais method

The Feldenkrais method consists of a system of ideas and principles concerning efficient and effective movement organization. It was developed by Moshe Feldenkrais in the second half of the 20th century (Feldenkrais, 1947, 1981), partly based on his extensive experience with the martial art of Judo (Feldenkrais, 1952). These principles are applied in movement lessons, which can be either taught verbally to a group of people or taught individually by guidance through manual touch. The Feldenkrais method is used by people of varying motor abilities and in a variety of settings, ranging from performing arts (Nelson, 1989; Schlinger, 2006) to rehabilitation (Ives and Shelley, 1998; Buchanan, 2012).

Most bodily actions require the coordination of multiple components of the sensorimotor system to realize specific action goals (e.g., reaching a target with the hand) while maintaining other systemic functions (e.g., balance, breathing). One assumption of the Feldenkrais method is that general improvement in movement organization can be achieved by clarifying the functional relation between components that need to be integrated in action. This can be approached, for instance, by exploring the coordination between different body parts, by systematically varying postural and balance constraints, by guiding somatosensory attention to different aspects of a movement (through verbal instruction or manual touch), or by mental imagery. Feldenkrais lessons often begin with small-amplitude and slow movements in order to enhance processing of sensory information and facilitate exploration of alternatives to habitual movement and perception patterns. This is in line with Nikolai Bernstein's theoretical work on motor learning: “Certainly, the most sensible and correct training would be organized in a way that combined a minimization of effort with a large variety of well-designed sensations and that created optimal conditions for meaningfully absorbing and memorizing all these sensations” (Bernstein, 1996, p. 181). It has been argued that the approach is consistent with general principles of sensorimotor learning (Connors et al., 2010). However, scientific evidence for the potential effectiveness of the Feldenkrais method remains limited (Ives and Shelley, 1998; Buchanan, 2012).

Active movement and perception play an important role for sensorimotor development, learning, and rehabilitation (Held and Hein, 1963; Krebs et al., 2003; Lotze et al., 2003; Berthouze and Goldfield, 2008; Iftime-Nielsen et al., 2012; Adolph and Robinson, 2015). The active role of the participant is evident in Feldenkrais group lessons, which are performed by the participant under verbal guidance, but less obvious in manual interventions, where it is the practitioner who initiates movements and manipulates the participant's body parts. Feldenkrais proposed that the students' nervous system becomes more actively involved in the interaction, relating the manipulation to self-generated action, if the practitioner attends to minute responses of the student, exploring which variations of a movement (e.g., in direction and orientation) connect most clearly to other body parts and create a harmonious movement among them. This idea is applied in the “artificial floor” manipulation investigated in the present study.

### The “artificial floor”

**Figure 1:**
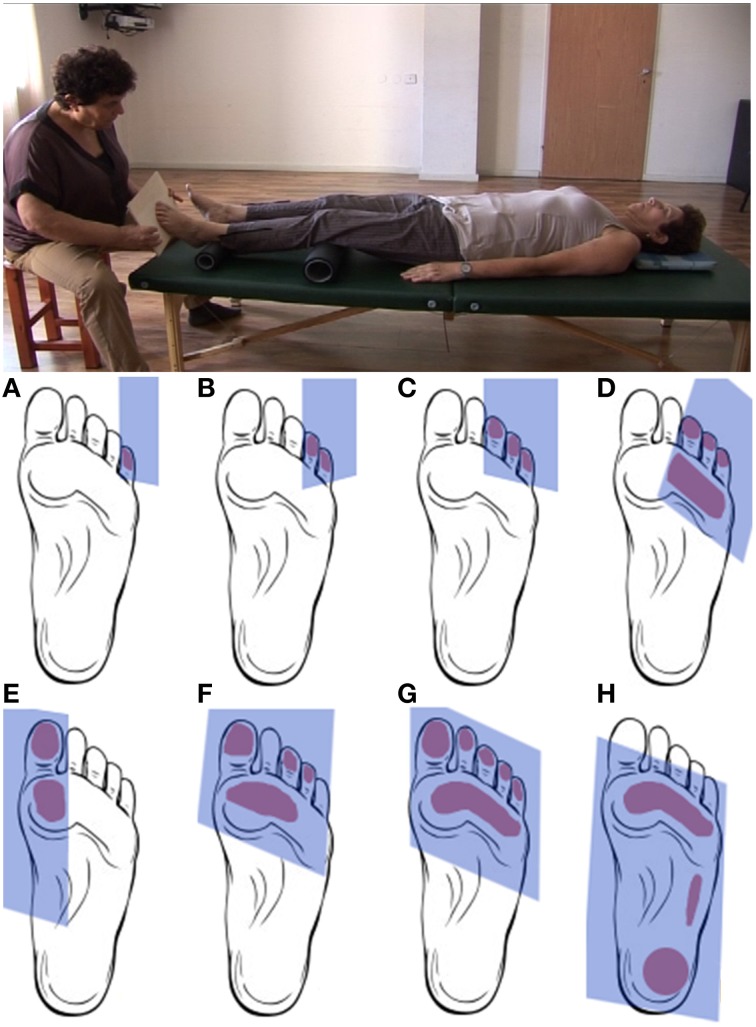
**Upper panel**. Positions of practitioner and participant during the artificial floor manipulation. *Lower panel*. Typical contact areas between board and foot during the artificial floor manipulation. The manipulation will typically proceed from touching and gently pushing individual or multiple toes **(A–C)** to gradually include larger parts of the foot sole **(D–G)** up to the entire foot **(H)**. Rather than being a simple tactile stimulation, the manipulation is individualized and interactive in the sense that the practitioner attends and adjusts to the response of the foot, potentially resulting movement in other body parts, as well as any other observable responses from the participant, for instance during the “return movement” when the contact is released. The present study compares two forms of the manipulation. In the *local* condition, the artificial floor is applied with the intention to explore mostly the mobility within the foot. The *global* intervention has a similar procedure, but the focus of the manipulation is on the connection and support from the foot to higher body parts. Exemplary videos of the Artificial Floor intervention can be found in the Supplementary Materials.

During the artificial floor manipulation (Feldenkrais, 1981, pp. 140–142), the participant is lying on the back, with the legs comfortably supported by rollers, while the practitioner touches one foot by means of a planar board (Figure 1, video in Supplementary Material). With the participant's legs positioned on the rollers, a small pressure applied to the toes or different parts of the sole of the foot, can create a *local* movement within the foot (the toes, the metatarsals, the ankle), but the manipulation can also have a more *global influence*, reaching other body parts via legs and spine, such as the pelvis, trunk, head and shoulders. Whether and how movement is transferred to different body parts, depends on the stimulation (contact point, direction, amplitude) and the neuromuscular state of the body (e.g., activation of muscles). Importantly, the goal of the practitioner is not to induce substantial movement in the foot or along the body, but to provide the participant's nervous system with sensory information about the *connection* from the foot to the rest of the body and, ultimately, with a sensation of the foot's function for body support under various orientations and contact points of the support surface. To achieve this, the practitioner adjusts the direction, timing and applied force to the configuration of the participant's foot and other body parts, as well as to the participant's responses to the stimulation.

Application of the artificial floor in a complete Feldenkrais lesson usually proceeds from a more *local* exploration of the mobility and anatomical relationships within the participant's foot to a more *global* mode, in which the practitioner explores the connection from the foot to the rest of the body, focusing on the function of the foot for body support. Presumably, differences in the attentional focus and intention of the practitioner lead to subtle differences in contact points as well as force directions and amplitudes during the intervention. These physical differences are not the subject of the present study and may indeed be difficult to define quantitatively across participants, as they are not pre-determined but develop individually and interactively during the manipulation, as described above. However, we hypothesized that the two forms of the manipulation induce distinct effects at the neural level which may be relatively invariant across participants due to the practitioner's invariant intentional focus (*local* vs. *global*) guiding the interactive manipulation. It should be noted that both forms of the artificial floor are typically used in Feldenkrais lessons, not as separate interventions but in combination. The distinction between the *local* and *global* manipulation is made in the present study in order to elucidate the role of the practitioner's attention to functional relationships between body parts.

### Neuroscientific background

The artificial floor manipulation reverses the usual relation between body and support surface: it is not the feet that “look for support” from the floor, but a “support surface”—a planar board moved by the practitioner's hands—that approaches and makes physical contact with the feet. Feldenkrais assumed that this allows the participant to experience the use of the foot for body support under various conditions of contact area and orientation, thereby eliciting a learning process that may influence subsequent use of the foot in standing and walking. Two major, non-trivial assumptions are that this is possible even though the participant is lying supine, without need for active postural stabilization, and despite the fact that the forces applied to the foot during the manipulation are much smaller than forces during bipedal standing or walking.

Tactile perception and proprioception from foot and ankle play a crucial role in stabilization of upright posture (Kavounoudias et al., 1998, 2001; Maurer et al., 2006; Wright et al., 2012), but it is a priori not evident to what extent sensory information provided in a lying position—such as during the artificial floor manipulation—will be interpreted by the nervous system in terms of balance control. It has been shown that neural activity in cortical and sub-cortical brain regions during proprioceptive ankle stimulation in a supine lying position in the magnetic resonance (MR) scanner correlates with balance performance (Goble et al., 2011). Moreover, mental imagery of standing or walking while lying in an MR scanner has been found to activate functionally plausible brain regions, including premotor and supplementary motor areas, basal ganglia and the cerebellum (Malouin et al., 2003; Jahn et al., 2004). These findings suggest that the nervous system can relate to balance and gait even in the physically constrained conditions determined by the MR scanner, in particular, this appears to be possible in a supine position.

Primary sensorimotor cortices are somatotopically organized along the central sulcus, with upper extremities and trunk represented more laterally and lower extremities represented in the medial wall (Penfield and Boldrey, 1937; Lotze et al., 2000; Zeharia et al., 2012). Brain activity in sensorimotor areas has been found to be less lateralized for the lower compared to the upper limbs (Kapreli et al., 2007). Relatively isolated foot movements (at the ankle joint) induce activity in medial sensorimotor cortex as well as higher-order motor areas, such as the supplementary motor area (SMA), premotor cortex and cingulate motor areas (CMA) (Dobkin et al., 2004). Comparing active to passive as well as electrically stimulated ankle movement, Francis et al. (2009) found increased activity in SMA, premotor cortex, dorsolateral prefrontal cortex as well as CMA for self-generated compared to externally generated movements. SMA and CMA were also found to be activated during preparation of active ankle movements compared to anticipation of passive movement (Sahyoun et al., 2004). These results indicate that, similar to upper-limb movements, primary sensorimotor cortices are activated even during externally-generated foot movements, while activity in higher-order motor areas (e.g., SMA and CMA) is related to preparation and performance of self-generated action.

Close links between action and perception have been postulated early in the history of experimental psychology (Lotze, 1852; James, 1890) and are corroborated by more recent behavioral and neuroimaging studies establishing bi-directional associations between movements and their sensory consequences (Greenwald, 1970; Prinz, 1990; Hommel et al., 2001; Kühn et al., 2010). Thus, neural action representations can be activated in the nervous system by a sensory stimulation that shares features with the sensory consequences of that action. To our knowledge, this has mostly been investigated using visual or auditory sensory stimuli (Brass et al., 2000; Paulus et al., 2012; Verrel et al., 2014). The physical stimulation of the foot in the artificial floor intervention is very different from the stimulation during actual standing, both in terms of amount of force and contact area. However, the *global* manipulation (if successful) shares an essential feature with the use of the foot for body support, as it aims to generate sensory information emphasizing the connection from the foot to other body parts. As a consequence of bi-directional action-perception links, we expected the *global* manipulation to elicit a corresponding neural action pattern (of using the foot to support the body) in the participant's nervous system.

Resting state fMRI offers the possibility to study spontaneous brain activity in the absence of an instructed task. It may therefore be especially appropriate to investigate potential changes in neural dynamics induced by behavioral or neural interventions (Guerra-Carrillo et al., 2014). For instance, short-term effects of a tactile (comparing “real” to sham acupuncture) intervention have been demonstrated on functional connectivity during resting state (Dhond et al., 2008). Also, extensive practice of new sensorimotor tasks influences spontaneous brain activity in functionally specific ways (Albert et al., 2009; Taubert et al., 2011; Vahdat et al., 2011). Finally, resting state activity has also been shown to be related to subjective experience (in this case, amount of unwanted thought) during the measurement (Kühn et al., 2013). Resting state analysis is therefore promising to study neural correlates of potential short-term effects of the artificial floor intervention.

### The present study

Anecdotal evidence suggests that the artificial floor intervention described above can have short-term effects on participant's use and subjective experience of the feet and rest of the body, such as a clearer contact to the floor and support/push from the foot to the head during standing and walking directly after the intervention. As the intervention itself can hardly induce any peripheral changes (e.g., in muscle-tendon length, muscle force), such effects would have to be due to changes in sensorimotor organization at the level of the central nervous system. The aim of the present exploratory study is to investigate these hypothesized neural changes in terms of brain activity in a functionally related foot-pushing-task as well as during resting state. In addition, we hypothesized that a subtle variation in the application of the artificial floor affects the way in which the manipulation engages the participant's nervous system and thereby influence subsequent neural activity during related motor tasks or during resting state. More specifically, we predicted that the *local* intervention, exploring the movement *within* the foot in response to touch at the toes and different parts of the foot sole, would mainly increase processing in brain areas representing that specific body part. In contrast, applying the artificial floor with a *global* focus on the motor function of body support, was hypothesized to engage broader and/or higher-level neural action representations.

To assess potential changes in neural organization following the two forms of the artificial floor manipulation (*local, global*) described above, we use fMRI to measure brain activity during a motor task mimicking the use of the foot for body support in a supine position (gently pushing with the foot onto a horizontal support surface) as well as resting state activity before and after each intervention (with the feet standing on the same support surface). The intervention was carried out by an expert Feldenkrais practitioner with more than 30 years of professional experience (one of the authors, EA). In order to minimize perturbation due to repositioning, the intervention and all tasks were carried out while participants were lying in the MR scanner. An independent localizer task before the experiment was used to determine regions of interest (ROIs) activated during the foot-pushing task. Based on the above reasoning, we predicted that application of the *local* artificial floor would lead to an increased activity in regions (both during pushing and resting state) in sensorimotor areas representing the stimulated foot. For the *global* artificial floor intervention, we predicted more widespread activity including higher-level motor areas, reflecting a more function/action-related neural processing.

## Methods

### Participants

Twenty-one participants (10 men, age range 19–30 years, mean age 24.8 years) took part in the study after written informed consent and approval of the Ethics committee of the German Psychological Society (DGPs). According to self-report, all participants were right-handed and did not have any history of neurological disorder, chronic pain, or medical conditions impairing movement or balance. Participants were also selected to have previously participated in MR studies, in order to minimize the likelihood of physical or emotional discomfort during the experiment. Prior to the study, participants were informed that the goal of the study was to investigate the link between perception and movement in tasks involving being touched at the foot sole and gently pushing the foot against the floor, respectively. No reference was made to the Feldenkrais method.

### Experimental protocol

A highly experienced and internationally recognized practitioner and teacher of the Feldenkrais method (one of the authors, EA) instructed participants for the active movement tasks, repositioned participants' legs between conditions, and carried out the intervention (“artificial floor”) in the scanner. Table 1 gives an overview of the experimental protocol. For the *functional localizer task* (6 min), performed at the beginning of the experiment, participants had both knees bent, the feet standing on a solid, horizontal support surface, positioned at the same vertical level as their body. Participants were (previously) instructed to repeatedly push the foot into the support surface and release again, performing this movement gently and with as little effort as possible while sensing any resulting somatosensory sensations along their body. The pushing movement was performed in 20-s blocks, randomized for the left and right foot, with 20-s resting periods between active blocks. Eyes were open during this task. The tasks were cued by visual stimuli, with a black screen denoting rest period and a green screen with centrally presented letter L (R) denoting pushing with the left (right) foot.

**Table 1:** **Overview of the experimental protocol in the MR scanner**.

**Condition**	**Leg position**	**Tasks**	**Duration (min)**
Functional localizer	Both feet standing	Push left, push right, rest	6
Left push *(baseline)*	Left foot standing, right leg long	Push left, rest	4
Resting state *(baseline)*	Both feet standing	Rest	3
Intervention *(local/global)*	Both legs long	Rest	3
Left push *(post-local/post-global)*	Left foot standing, right leg long	Push left, rest	4
Resting state *(post-local/post-lobal)*	Both feet standing	Rest	3
Intervention *(global/local)*	Both legs long	Rest	3
Left push *(post-global/post-local)*	Left foot standing, right leg long	Push left, Rest	4
Resting state *(post-global/post-local)*	Both feet standing	Rest	3

Subsequently, participants performed a push/release task (4 min, alternating 20-s blocks of push/release and rest) with only their left foot standing on the support surface (the right leg was long, supported by cushions and rollers for the participant's comfort). Participants were instructed to keep their eyes closed during this task in order to enhance somatosensory attention to the body (Marx et al., 2003). This task was organized in alternating 20-s blocks of activity and rest, cued by a white and black screen, respectively. The strong brightness contrast allowed the cues to be perceived even with closed eyes. This push/release task was followed by a *baseline resting-state* period (3 min), during which participants had both knees bent, with the feet standing on the support surface, and with the instruction to close their eyes and rest.

**Figure 2:**
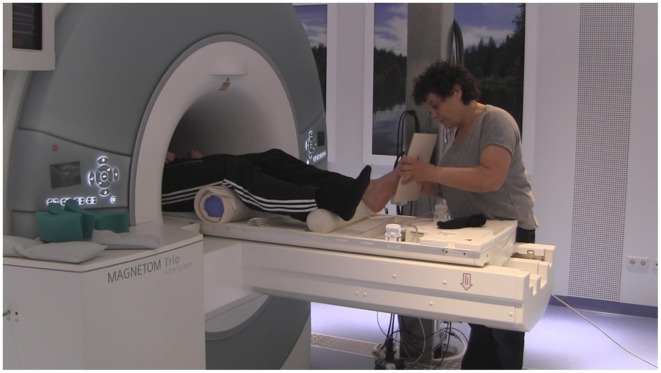
**Experimental setup, with the practitioner (EA) performing the artificial floor intervention in the MR scanner**.

During the artificial floor intervention (see Figures 1, 2, video in Supplementary Material), the participants' legs were both lying on rollers while the Feldenkrais practitioner gently touched the participant's bare left foot by means of a planar board (3 min). The participant's sock was removed from the left foot prior to each part of the intervention, in order to allow differentiated touch of the foot, and put back on directly afterwards to avoid cooling. During the *local* manipulation, the intention of the practitioner delivering the stimuli was to explore movement *within* the left foot, that is, touching and applying minimal force to toes, balls, or the whole sole of the foot, investigating the resulting movement in the toes, foot and ankle. During the *global* manipulation, the experimenter delivered comparable tactile stimuli through the board, but now exploring the connection from the foot to the rest of the body and emphasizing the role of the foot for body support. Each of the two forms of the intervention *(local, global)* was followed by the pushing task with the left foot (4 min) and the resting state task (3 min) described above. The order of intervention conditions (*local, global)* was counterbalanced across participants. The Feldenkrais practitioner was only informed about the order of the conditions (*local, global*) before the first intervention with each participant, such that her initial interaction with the participant was not influenced by this information.

The net scanning time was approximately 45 min. After the experiment, participants were informally interviewed about potential changes they experienced in the pushing movement after the intervention, during upright standing after the experiment (e.g., weight distribution and contact of the feet to the floor), any discomfort during the experiment, and any potential previous experience with the Feldenkrais method.

### Scanning procedure

Images were collected on a 3T Magnetom Trio MRI scanner system (Siemens Medical Systems, Erlangen, Germany) using a 32-channel radiofrequency head coil. The structural images were obtained using a three-dimensional T1-weighted magnetisation prepared gradient-echo sequence (MPRAGE) based on the ADNI protocol (www.adni-info.org) [repetition time (TR) = 2500 ms; echo time (TE) = 4.77 ms; *TI* = 1100 ms, acquisition matrix = 256 × 256 × 176, flip angle = 7°; 1 × 1 × 1 mm^3^ voxel size]. Functional images, both for the functional localizers and resting state analysis, were collected using a T2^*^-weighted echo planar imaging (EPI) sequence sensitive to blood oxygen level dependent (BOLD) contrast (*TR* = 2000 ms, *TE* = 30 ms, image matrix = 64 × 64, FOV = 216 mm, flip angle = 80°, voxel size 3 × 3 × 3 mm^3^, 36 axial slices).

### fMRI data pre-processing

The fMRI data were analyzed using SPM8 software (Wellcome Department of Cognitive Neurology, London, UK). For the functional analysis (pushing task), the first four volumes of all EPI series were excluded from the analysis to allow the magnetisation to approach a dynamic equilibrium. Data processing started with slice time correction and realignment of the EPI datasets. A mean image for all EPI volumes was created, to which individual volumes were spatially realigned by means of rigid body transformations. The structural image was co-registered with the mean image of the EPI series. Then the structural image was normalized to the Montreal Neurological Institute (MNI) template (resampling voxel size of 3 × 3 × 3 mm), and the normalization parameters were applied to the EPI images to ensure an anatomically informed normalization. Participants showing head motion above 3.5 mm of maximal translation (in any direction of x, y, or z) and 2.0° of maximal rotation during scanning would have been excluded (this was not the case for any participant). A spatial filter of 8 mm full-width at half maximum (FWHM) was used. Low-frequency drifts in the time domain were removed by modeling the time series for each voxel by a set of discrete cosine functions to which a cut-off of 128 s was applied.

For the resting state analysis, the first five volumes were discarded to allow the magnetisation to approach a dynamic equilibrium, and for the subjects to get used to the scanner noise. Part of the data pre-processing, including slice timing, head motion correction (a least squares approach and a 6-parameter spatial transformation) and spatial normalization to the MNI template, were conducted using the Data Processing Assistant for Resting State fMRI toolbox (DPARSF; Chao-Gan and Yu-Feng, 2010). A spatial filter of 4 mm FWHM was used. After pre-processing, linear trends were removed and the fMRI data were temporally band-pass filtered (0.01–0.08 Hz) to reduce low-frequency drift and high-frequency respiratory and cardiac noise (Biswal et al., 1995).

### fMRI data analysis

Brain activity during the pushing tasks was analyzed at the first (within-subject) level using regressors for *left* and *right* pushing blocks. Each block (20 s duration) was convolved with a hemodynamic response function and head movement parameters were included in the design matrix. We were interested in the contrast comparing left and right pushing (functional localizer) as well as left pushing compared to rest (functional localizer and pushing task during the experiment). Resting state activity was analyzed in terms of regional homogeneity. Based on the fact that fMRI activity is typically spatially clustered (Tononi et al., 1998), this analysis approach determines voxels at which BOLD fluctuates in synchrony with its neighboring voxels (Zang et al., 2004; Wu et al., 2007). The analysis was performed with the toolbox DPARSF (Chao-Gan and Yu-Feng, 2010), using Kendall's coefficient of concordance of the time series of a given voxel with those of its nearest 26 neighbors. ReHo was calculated within a brain-mask, which was obtained by removing the tissues outside the brain using the software MRIcron (https://www.nitrc.org/projects/mricron).

The resulting images were entered into a series of one-sample *T*-tests at the second (between-subject) level. For the functional localizer task, the resulting SPMs (*left-push* > *right-push, left-push* > *rest*) were thresholded at *p* < 0.05 with familiy-wise error correction (FWE). For the pairwise comparisons of resting state activity (ReHo) and functional activations in the pushing task before and after the intervention, the resulting statistical maps (*post-local* > *baseline, post-global* > *baseline, post-local* > *post-global, post-global > post-local*) were thresholded at *p* < 0.005 (uncorrected) with an additional cluster size threshold of *k* = 42. The required cluster size was determined by Monte Carlo simulation (AlphaSim; Ward, 2002) to ensure that the probability of type I error was not greater than 0.05. Reported coordinates correspond to the MNI coordinate system.

Regions of interest (ROIs) were defined based on brain activation during the functional localizer task at the beginning (*left-push* > *right-push, left-push* > *rest*). Mean ReHo values in the three conditions (*baseline, post-local, post-global*) in these ROIs were extracted using MarsBaR toolbox (Brett et al., 2002) and compared using paired *T*-tests.

## Results

**Figure 3:**
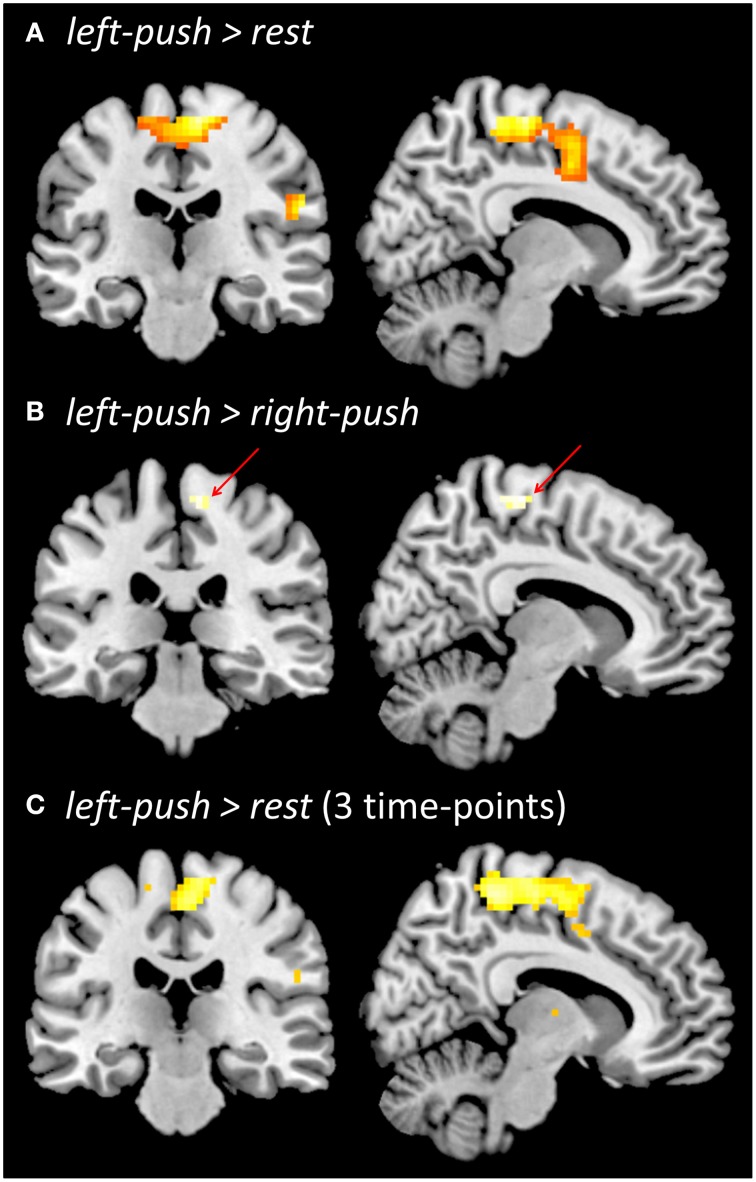
**Functional brain activation during pushing one foot onto the horizontal support surface. (A)** Localizer prior to experiment, *left-push > rest*. **(B)** Localizer prior to the experiment, *left-push > right-push*. **(C)** During the experiment, across three time-points (baseline, post-local, post-global), *left-push > rest*. All contrasts are thresholded at *p* < 0.05, FWE.

**Figure 4:**
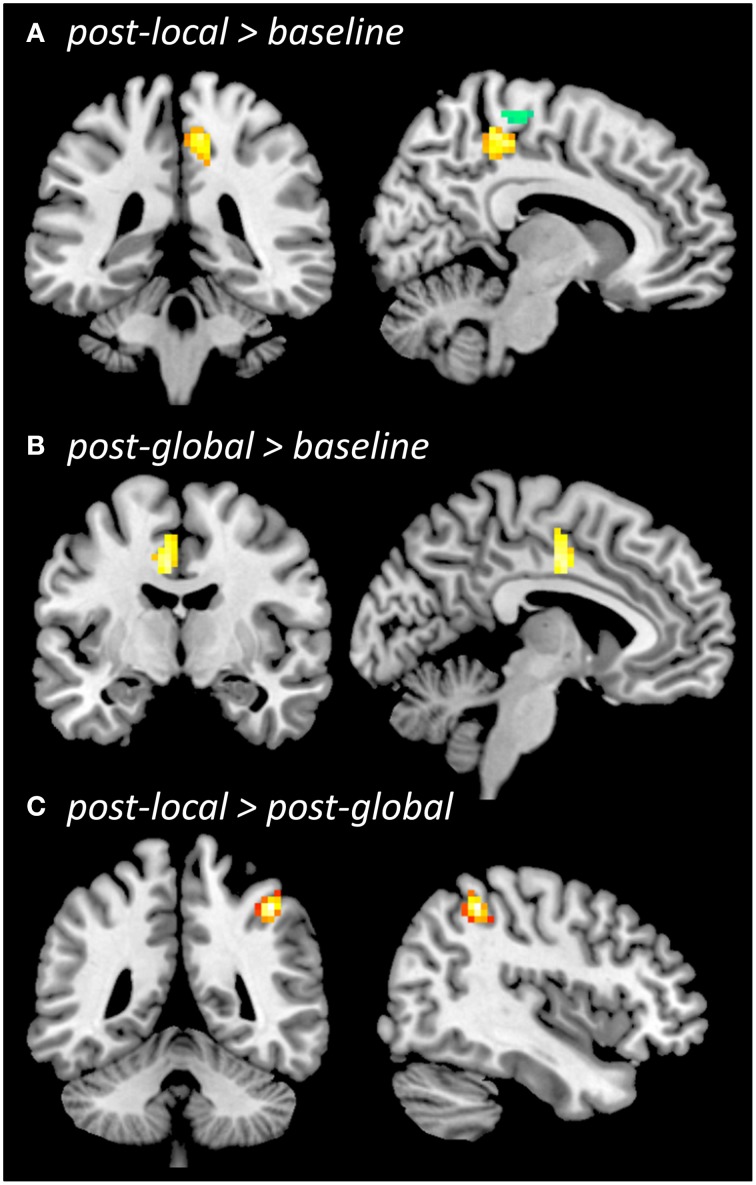
**Regional homogeneity (ReHo) during resting state**. Whole-brain comparison *post-local > baseline* (**A**, overlaid with functional localizer ROI, *left-push > right-push*), *post-global > baseline* **(B)**, *post-local > post-global* **(C)**, all thresholded at *p* < 0.005 (uncorrected), cluster size *k* = 42. The contrast *post-global > post-local* (not shown) did not reveal significant differences.

Significant brain activity during pushing (during localizer or during experiment) and differences in ReHo between conditions are shown in Figures 3, 4, and reported in more detail below. Detailed results are only reported for primary and higher-order sensorimotor brain regions. Thresholded SPMs for all contrasts resulting in statistically significant effects are available as Supplementary Material for further inspection.

### Functional activity during pushing

Push-related activity in the functional localizer prior to the experiment was analyzed in two ways. Comparing *left-push* to *rest* (Figure 3A), we found a large significant cluster in medial sensorimotor cortex with peak activation in right medial motor cortex (*p* < 0.05, FWE; 15, −34, 64; 543 voxels; *Z* = 6.37) as well as more lateral clusters (see Supplementary Material). Comparing *left-push* to *right-push* (Figure 3B) revealed a significant cluster in right medial motor cortex (*p* < 0.05, FWE; 16 voxels; 9, −25, 64; *Z* = 5.05).

Aggregating pushing-related activations (*left-push* > *rest*; Figure 3C) across the three time points of the experiment *(baseline, post-local, post-global)* shows similar activation to the functional localizer at the beginning of the experiment, peaking in right medial motor cortex (*p* < 0.05, FWE; 3, −19, 61; 492 voxels; *Z* = 5.79). Comparison of push activation *(left-push > rest)* for each pair of time points showed a significant cluster in the occipital lobe for *post-local > post-global* (*p* < 0.005, *k* ≥ 42; −24, −82, 16; 51 voxels; *Z* = 3.36) and in the left temporal lobe for *baseline > post-local* (*p* < 0.005; *k* = 42; −60, −1, −2; 49 voxels; *Z* = 3.19). A ROI-based analysis (based on functional localizer prior to the experiment, *left-push > rest, left-push > right-push*) did not reveal any significant differences of the activation during pushing between the three time points (*baseline, post-local, post-global*).

### Resting state analysis (ReHo)

ReHo increased from *baseline* to *post-local* (Figure 4A) in right medial sensorimotor cortex (*p* < 0.005, *k* = 42; 6, −33, 51; *Z* = 3.65; 62 voxels) as well as additional clusters in temporal cortices (see Supplementary Material). The ROI-based analysis showed that ReHo increased in the ROI defined by the functional localizer prior to the experiment [*left-push > rest*: *t*_(20)_ = 2.46, *p* = 0.011; *left-push > right-push*: *t*_(20)_ = 1.49, *p* = 0.076] as well as the activation in the push-trials during the experiment [*left-push > rest*: *t*_(20)_ = 2.52, *p* = 0.01). Decreases in ReHo from *baseline* to *post-local* were found in lateral sensorimotor areas as well as several additional brain areas (see Supplementary Material).

ReHo increased from *baseline* to *post-global* (Figure 4B) in left SMA/CMA (*p* < 0.005, *k* ≥ 42; −3, −9; *Z* = 3.40; 44 voxels), as well as in additional clusters in left temporal cortex (see Supplementary Material). This increase was significant in one of the ROIs defined by the functional localizer [*left-push > rest*: *t*_(20)_ = 1.8, *p* = 0.043; but *left-push > right-push*: *t*_(20)_ = −0.16, n.s.] and marginally significant in the ROI defined by the pushing-related activation across the three time points during the experiment [*left-push > rest*: *t*_(20)_ = 1.48, *p* = 0.077]. Decreases in ReHo from *baseline* to *post-global* were found in lateral sensorimotor areas (see Supplementary Material).

Comparing ReHo between *post-global* and *post-local* (Figure 4C) showed a significant cluster in right lateral motor cortex (*post-local > post-global*; *p* < 0.005, *k* = 42; 42, −45, 51; *Z* = 4.74; 46 voxels) and one additional cluster in right temporal cortex (see Supplementary Material). The difference was marginally significant in the ROIs defined by the functional localizer [*left-push > rest*: *t*_(20)_ = 1.52, *p* = 0.072, *left-push > right-push*: *t*_(20)_ = 1.67, *p* = 0.055] and significant in the ROI defined by the push-activation across the three time points during the experiment [*left-push > rest*: *t*_(20)_ = 1.96, *p* = 0.032]. The opposite contrast (*post-global > post-local*) did not show any significant differences in the whole-brain or ROI-based analyses.

### Subjective reports

None of the participants reported prior experience with the Feldenkrais method. One participant experienced discomfort while lying in the scanner. Data from this participant (not included in the *N* = 21 above) were excluded from all analyses.

About half of the participants reported changes in subjective experience during performance of the pushing movement after the artificial floor intervention. Among other things, participants described that their “foot felt more relaxed, resulting in more pushing with the ball of the foot rather than just the heel,” that “the pushing felt easier,” that “it was easier to control the movement,” that “the contact area of the foot on the support surface was larger,” or that they “generally felt more relaxed” after the intervention. (No distinction was made in the informal interview between the *local* and the *global* intervention). Moreover, several participants reported differences between the left and the right leg during bipedal upright standing (outside the scanner) after the experiment. For instance, participants described the left foot and leg as feeling “lighter but in clearer contact with the floor,” having a “wider contact area,” feeling “more relaxed” or “more stable, better able to keep balance,” compared to the right side.

## Discussion

We set out to study potential changes in neural activity during a gentle foot-pushing task (related to body support) and resting state, induced by two forms of the artificial floor intervention: the *local* manipulation, in which the practitioner focuses on mobility within the foot, and the *global* manipulation, in which the practitioner explores the connection from the foot to the rest of the body, focusing on the function of the foot for body support. Both forms of the intervention were carried out with the high interactive quality described in the Introduction, attending and adjusting to minute cues and responses the practitioner perceives visually or haptically during the manipulation. However, the *global* intervention was hypothesized to address a more complex and action-related function (body support) than the *local* intervention (mobility within the foot). We did not observe reliable changes in pushing-related activity in sensorimotor brain regions from pre- to post-intervention. However, resting state activity (quantified by regional homogeneity, ReHo), changed in primary and higher-order motor regions in distinct ways for the two forms of the intervention.

### Brain activity while pushing with the foot

Brain activity while gently pushing the foot onto a horizontal support surface (and releasing the push) was measured prior to and during the experiment, as an active motor task hypothesized to be functionally related to the artificial floor intervention. Brain activity was comparable for left-foot pushing across these conditions, including broad areas of primary sensorimotor cortices and higher-level motor areas. Contrasting left- to right-push showed significant differences in right medial primary motor cortex, consistent with somatotopic representation of the foot (Penfield and Boldrey, 1937; Lotze et al., 2000). Brain activity in the pushing-task was used to define ROIs, in order to assess and compare potential changes in brain activity induced by the interventions.

Brain activity during the push task in sensorimotor cortices or in the ROIs defined prior to the experiment was not reliably affected by the artificial floor intervention. Thus, the fMRI measurement does not provide evidence that one or both of the two interventions altered the neural representation of self-generated pushing. In particular, we did not observe the hypothesized more widespread activity after the global intervention. At the same time, the absence of an effect suggests that the pushing action did not reliably differ between the different time points. Hence, differences between conditions found in resting state activity (which was measured after the pushing task) cannot be portrayed as mere after-effects of different ways of active pushing, but instead as consequences of the artificial floor intervention.

### Effects of artificial floor on resting state activity

Significant increases in resting state activity in sensorimotor and higher motor areas were observed after the artificial floor intervention. Moreover, changes from *baseline* (pre-intervention) to *post-local* and *post-global* differed in spatial location. The *local* intervention induced an increased ReHo in right medial primary motor cortex, consistent with somatotopic foot representations found in previous studies (Penfield and Boldrey, 1937; Lotze et al., 2000). In contrast, the *global* intervention induced significant ReHo increases in left SMA/CMA. While these regions are also part of the medial motor regions, activation was more anterior after the *global* intervention than after the *local* intervention, and in the opposite hemisphere. Directly comparing ReHo post-global and post-local showed greater ReHo after the *local* intervention in right lateral primary motor cortex. ReHo in pushing-related ROIs increased relative to *baseline* both after the *local* and the *global* intervention, and the increase tended to be more pronounced *post-local*.

These findings are partly consistent with our predictions. The *local* intervention induced an increase in ReHo in the primary motor cortex representation of the stimulated foot. This suggests a relatively confined, indeed “local,” effect of this intervention, which may or may not be specific to the particular technique. For instance it might be a general sensorimotor attention effect, that could potentially also be induced by other forms of tactile stimulation. In contrast, the increase from *baseline* to *post-global* was not localized in primary motor cortex (as for the local intervention) but in a more anterior part of motor cortex (SMA, CMA) that has been related to higher-level aspects of motor control (Sahyoun et al., 2004; Francis et al., 2009). Moreover, this increase in ReHo was found in the left (ipsilateral) hemisphere, unlikely to be activated by plain tactile stimulation of the left foot. However, directly contrasting resting state activity after the two interventions did not reveal regions with greater ReHo *post-global* relative to *post-local*. Clearly, the processes underlying these changes in resting state activity remain to be investigated in more detail. Yet, the results are in general compatible with the prediction that the *global* intervention would engage more functional, action-related brain networks, while the *local* intervention would engage brain regions representing the stimulated body part.

### Limitations, strengths, and outlook

As, to our knowledge, elements of the Feldenkrais method have never been investigated neuroscientifically before, this was a highly exploratory study. Yet, the study was aimed at a relatively subtle effect, namely the (hypothesized) differential effect of two ways of performing the artificial floor intervention: *local*, exploring small movements of foot and ankle as a consequence of the touch; and *global*, focusing on the function of the foot for body support. As the study investigated this subtle difference, no “non-Feldenkrais” control condition involving tactile foot stimulation was included in the study design. As a consequence, we cannot rule out the possibility that increased ReHo in primary sensorimotor areas after foot stimulation, as found after the *local* intervention, might be an effect of increased somatosensory attention to the foot (Johansen-Berg et al., 2000).

Unfortunately, our experimental setup did not allow for measurements of the movement of the board, the contact forces and area between board and foot, or the resulting (minimal) movement in the participant's body. In particular, we are unable to report potential differences in these parameters between the *local* and *global* intervention. We can therefore not rule out the possibility that observed differences in ReHo may be explained by systematic differences in low-level parameters of the intervention. Also, the push-forces exerted by the foot on the floor in the active conditions, could not be recorded in the scanner, and we did not assess potential changes in movement patterns after the intervention outside the scanner. Thus, further research is needed to characterize the artificial floor intervention in terms of the physical interaction between board and participant's foot as well as to assess potential effects of the intervention at the motor-behavioral level.

Several methodological strengths of the present study should also be pointed out. Changes in resting state activity were present in ROIs defined by independent functional localizer tasks. While differences in ReHo between conditions were only found with relatively liberal significance thresholds (*p* < 0.005, uncorrected), probability of type-I error was controlled for by requiring an appropriate minimal cluster size determined by Monte Carlo simulation (Ward, 2002). All participants had previous experience with MR studies in order to minimize potential discomfort or distress. Participants were interviewed about potential discomfort after the experiment and the single participant reporting discomfort was excluded from the analysis. Participants had no prior experience with the Feldenkrais method (according to self-report after completion of the experiment) and were naive to the goals of the study. The intervention was carried out by an expert Feldenkrais practitioner (EA). The practitioner was only informed directly before the first intervention about the sequence of conditions to avoid effects this knowledge might have on the initial interaction with the participant.

The artificial floor represents some principles general to the Feldenkrais method, in particular the individualized and interactive nature of manipulations and the focus on functional relationships. The results of the present study indicate that differences in focus on the side of the practitioner, even in the relatively abstract interaction of touching the participant at the foot by means of a planar board, are associated with reliable differences in neural resting state activity. Moreover, despite the fact that the present study used a very minimalistic intervention in a highly constrained experimental setup, several participants reported subjective changes such as a more “relaxed” foot, a larger contact area of the foot on the support surface, increased ease of the pushing movement, or more stability in standing on the leg. While these reported effects are confounded with task repetition, they were mostly specific to the left side (to which the intervention applied) and are consistent with anecdotal reports of experienced practitioners about frequently observed effects of the artificial floor intervention. Typical Feldenkrais sessions last longer (e.g., 30–45 min) than the intervention used here (2 × 3 min) and involve more body parts and functional relationships between them. For instance, a Feldenkrais session involving the artificial floor manipulation would generally connect the support/push function of the foot more explicitly to the rest of the body, for instance by asking participants to perform related active movements by themselves (such as the pushing movement used in the present study, or getting up to walk a few steps) or by providing additional manual touch and guidance at other parts of the body. Further, studies are needed to investigate potential effects of more complete Feldenkrais interventions on the nervous system as well as subsequent motor behavior.

## Conclusions

The results of this exploratory study show that a short, non-intrusive sensorimotor intervention based on the Feldenkrais method can have effects on spontaneous cortical activity in functionally related regions. Moreover, two variants of performing the artificial floor manipulation, focusing on either foot mobility (*local)* or functional use of the foot for body support (*global)*, differentially affected subsequent resting state activity. Increased resting state activity in higher-order motor areas supports the hypothesis that the *global* intervention engages action-related neural processes.

### Conflict of interest statement

One of the authors (EA) is a Feldenkrais practitioner and teacher, directing professional training programmes in the Feldenkrais method. One of the authors (JV) took part in one such training but is not working as a practitioner. The other authors are not associated in any way with the Feldenkrais method. The current study does not address the effectiveness as such but investigated the short-term influence of a brief sensorimotor manipulation motivated by the Feldenkrais method. We therefore do not see any actual conflict of interest.

## Supplementary material

Video S1 **Videos of a Feldenkrais practitioner (EA) performing the artificial floor intervention**.

Video S2 **Thresholded statistical parametric maps for the different second-level analyses, for activation during pushing and during resting state (ReHo)**.
